# Analysis of factors influencing casual sexual behavior among male college students in Zhejiang Province, China

**DOI:** 10.1371/journal.pone.0250703

**Published:** 2021-05-03

**Authors:** Zhongrong Yang, Weiyong Chen, Meihua Jin, Wanjun Chen, Lin Chen, Xin Zhou

**Affiliations:** 1 Huzhou Center for Disease Control and Prevention, Huzhou, Zhejiang Province, China; 2 Department of HIV/STD Control and Prevention, Zhejiang Provincial Center for Disease Control and Prevention, Hangzhou, Zhejiang Province, China; The Chinese University of Hong Kong, HONG KONG

## Abstract

**Objective:**

The purpose of this study was to explore the situations and factors influencing casual sexual behavior among male college students, in order to provide scientific evidences and measures of the prevention and control for HIV/AIDS.

**Method:**

Using the stratified cluster sampling method, male college students who self-reported sexual behavior were selected as survey subjects in 13 colleges and universities in 11 cities of Zhejiang Province from October to November 2018. We used a custom online questionnaire to collect information on the demographic characteristics, sexual attitudes, sexual behaviors, and HIV interventions of the respondents. The χ^2^ test was performed on the composition ratios between different groups. With the occurrence of casual sexual behavior as the dependent variable, logistic regression was used to analyze the factors influencing casual male sexual behavior.

**Results:**

A total of 2734 male college students were surveyed, aged 20.20±1.41 years, of which 595 had casual sex, accounting for 21.7%. The rate of HIV prevention awareness among the participants was 85.1%. Multivariate analysis showed that receiving a self-assessment of HIV risk conducted by the school (*Ajusted OR* = 1.45, 95% *CI* = 1.14–1.84), knowing that HIV self-test kits were available at school (*Ajusted OR* = 2.02, 95% *CI* = 1.56–2.62), accepting one-night stands (*Ajusted OR* = 2.82, 95% *CI* = 2.18–3.66), accepting commercial sex (*Ajusted OR* = 1.95, 95% *CI* = 1.53–2.48), being a man who has sex with men (*Ajusted OR* = 1.81, 95% *CI* = 1.37–2.39), being a senior (*Ajusted OR* = 0.46, 95% *CI* = 0.30–0.71), having knowledge of HIV/AIDS prevention and treatment (*Ajusted OR* = 0.66, 95% *CI* = 0.51–0.86), and knowing that the CDC provides HIV testing services (*Ajusted OR* = 0.56, 95% *CI* = 0.41–0.77) were factors influencing male college students’ casual sexual behavior.

**Conclusion:**

Male college students who have causal sexual behaviors have a high degree of openness in sexual attitudes, insufficient knowledge of AIDS prevention, and knowledge of HIV testing-related information but low testing rates. For male college students’ HIV prevention education intervention, it is necessary to emphasize the establishment of correct sexual attitudes and concepts and promote safe sexual behaviors to prevent the spread of HIV.

## Introduction

As a major global infectious disease, AIDS is currently a serious social problem in most countries [1–3]. In recent years, the HIV/AIDS epidemic among Chinese students has risen rapidly, and they have become a key population for HIV prevention and treatment [4]. The most common way to transmit HIV infections is through unprotected sexual behaviors [5]. The AIDS epidemic assessment and sentinel surveillance in Zhejiang Province showed that in that province, HIV is mainly spread by men who have sex with men (MSM), heterosexual commercial sex and heterosexual noncommercial sex, and the HIV infection rate among MSM has reached 8.6%, exceeding the WHO 5% AIDS epidemic warning threshold [6–8].

Many studies had reported that nearly half of college students had casual sexual behaviors during the university study [9–11], casual sexual behaviors may cause unexpected pregnancy or affect their studies [12, 13]. Some studies have carried out researches on the casual sexual behaviors of college students and whether they drink alcohol, and the results found that drinking alcohol may increase the occurrence of casual sexual behaviors to a certain extent [11, 14]. With the common occurrence of premarital sex among college students, the age of their first sexual encounter will be earlier, and the proportion of unprotected sexual contact will increase, which will increase the risk and impact of HIV transmission [15]. Therefore, the prevention of HIV/AIDS among male college students who engage in casual sexual behaviors cannot be delayed.

However, there are few studies on the situations and factors influencing casual sexual behavior among male college students, and this study will fill this gap. This research was a cross-sectional survey of casual sexual behaviors among male college students from October to November 2018. It explored the current situation and influencing factors of casual sexual behavior among male college students. The intent is to provide a basis for colleges and universities to develop targeted prevention and control procedures for HIV and other sexually transmitted diseases (STDs).

## Materials and methods

### Participants and settings

Zhejiang Province is located in the Yangtze River Delta region on the southeast coast of China. It is between 118°01’~123°10’ east longitude and 27°02’~31°11’ north latitude. In 2019, the Zhejiang Provincial Department of Education and the Zhejiang Provincial Health Commission jointly issued a document requiring schools to include AIDS education in their health education plans, organize the implementation of preventive education, and encourage college students to take the initiative to receive voluntary AIDS counseling and testing services. A survey was conducted among students attending 13 colleges in 11 districts and cities in Zhejiang Province from October to November 2018, including 3 colleges/universities in Hangzhou and 1 college/university each in another 10 cities. The types of universities were chosen as university or vocational technical college, and the selection of universities was as recommended by the local CDC. A total of 31,674 students were surveyed, of which 14,320 were male students. The inclusion criterion for the study subjects resulting in the selection of 2734 male students was that they self-reported having sex, accounting for 19.1% of the total number of male students. This study complies with the Declaration of Helsinki, which was reviewed and approved by the Ethics Committee of the Zhejiang Provincial Center for Disease Control and Prevention as well, and for which the batch number is 2018–036. All survey participants provided signed informed consent.

### Study variables and measurements

The questionnaire was formulated after reading the domestic and foreign literature, discussions with the research team, and conducting preliminary surveys of students. The main content of the questionnaire included general population sociological characteristics, knowledge of HIV and STDs, the occurrence of sexual behaviors, and the acceptance of interventions. There are 7 items about HIV/AIDS prevention and treatment knowledge, where answering 5 or more items correctly was defined as having HIV/AIDS prevention and treatment knowledge. These 7 items were as follows: Whether knowing AIDS is an incurable and serious infectious disease? (Item 1); Whether the main mode of HIV transmission among young Chinese students is male homosexual sex?(Item 2); Whether it can be judged by appearance that a person is infected with HIV?(Item 3); Whether daily life and contact spread HIV?(Item 4); Whether the correct use of condoms reduces the risk of infection and transmission of HIV?(Item 5); Whether there is a need to proactively seek HIV counseling and testing after high-risk sex?(Item 6); Whether knowing that the local government provides free antiretroviral treatment for people living with HIV/AIDS? (Item 7). Casual sexual behaviors means whether the participants ever had temporary sex (such as one-night stand / dating, sex with ordinary acquaintances) in the past year.

### Data collection

Using the stratified cluster sampling method, a random number table was used to select 3 departments in each university, and then classes were selected from each department by grade. An electronic questionnaire was generated with the web link or QR code, and was distributed to each participant by WeChat. The investigators were composed of tutors who were responsible for tutoring these classes, and were trained by the research team before the investigation.

### Quality control

Using the cross-sectional survey method, the students were organized by the teachers to fill in the online electronic questionnaires. Students who were currently not at school had the questionnaire network link sent to them, and they filled out the survey after being given the instructions. The measurement of condom use effectiveness included three questions as follows: Do you have confidence in discussing condom use with your partner before having sex? When the partner refuses to use a condom or does not carry a condom, is there any confidence not to have sex? Do you have the confidence to prepare condoms in advance before having sex? There were 5 options for answering these three questions: very confident, somewhat confident, confident, unconfident, and very unconfident, assigned scores of 3, 2, 1, 0, and −1, respectively. The measurement scores were divided into three groups, namely, unconfident, confident, and very confident, according to 1 point or less, 2–5 points and 6–9 points, respectively. The measured Cronbach’s alpha coefficient in this study was 0.852.

The investigators consisted of professionals from the local Center for Disease Control and Prevention and counselors from the middle classes selected by the universities. After unified training, a unified questionnaire was used to conduct an anonymous survey. The investigator explained the purpose, significance, investigation method, and privacy protection policy of the research before the survey and placed these descriptions in the opening remarks of the questionnaire. The research subjects were told that the purpose of the survey was to formulate strategies for protecting students from HIV and other STDs. The survey analyzed only data from this group, excluding personal data.

### Statistical analysis

SPSS version 21.0 software (IBM, Armonk, NY, United States) were used for data analysis. Some variables such as age, grade, hometown, household registration, family relationship, and sexual behavior characteristics are expressed by the composition rate. The *χ*^*2*^ test was used to compare the demographic characteristics of the male college students with different sexual behaviors. The measurement data are expressed by the mean ± standard deviation. Whether the participants engaged in casual sexual behavior was the dependent variable. Independent variables included demographic characteristics, sexual attitudes, acceptance of interventions, and self-efficacy of safe sexual behaviors. The single-factor logistic regression method was used to analyze the factors influencing casual sexual behavior among male college students. Variables with *P*<0.1 in the univariate analysis and demographic characteristics such as age, grade and hometown were included as independent variables in the multivariate logistic regression analysis model. *P*<0.05 was considered statistically significant.

## Results

### General characteristics

Among the 2734 subjects, 595 engaged in casual sexual behavior, accounting for 21.8%; 2139 engaged in no casual sexual behavior, accounting for 78.2%. Their ages were between 16 and 28 years old, with that of the casual sexual behavior group being 20.10±1.42 and that of the without casual sexual behavior group being 20.23±1.40. There were no significant differences in age, household registration, or family relationship between the two groups (*P*>0.05), but significant differences were found between the two groups for grade and hometown (*P*<0.05). The statistical analysis results are shown in Table 1.

**Table 1:** Demographic characteristics of 2734 male college students.

Variables	Casual sexual behavior group (n = 595)		Without casual sexual behavior group (n = 2139)		*x*^*2*^	*P*
	***n***	***%***	***n***	***%***		
**Age (years)**						
≤19	196	32.9	631	29.5	3.404	0.182
20–21	309	51.9	1135	53.1		
≥22	90	15.2	373	17.4		
**Grades**						
Freshman	121	20.3	405	19.0	21.072	<0.001
Sophomore	200	33.6	642	30.0		
Junior	206	34.6	674	31.5		
Senior	68	11.5	418	19.5		
Household registration^*^						
Zhejiang Province	437	73.6	1492	69.8	3.262	0.075
Other Provinces	157	26.4	647	30.2		
**Hometown**						
City	166	27.9	518	24.2	14.821	0.001
Town	109	18.3	292	13.7		
Rural area	320	53.8	1329	62.1		
**Family relationship**						
Harmonious	470	79.0	1639	76.6	1.479	0.224
General/disharmonious	125	21.0	500	23.4		

*There is missing data for one participant.

### Awareness rate of HIV prevention knowledge

Among the 2734 male college students, 2328 answered 5 questions correctly, accounting for 85.1%. Among them, the rate of correctly answering "Item 2" was higher in the casual sex group (49.41%) than in the noncasual sex group (43.43%), and there was a significant difference (*P*<0.05). The rate of correctly answering the other 6 questions in the two groups was between 63.19% and 97.34%. The rate of correctly answering "Item 3", "Item 4", "Item 5", and "Item 6" in the noncasual sexual behavior group was higher than that in the casual sexual behavior group, and the difference between the two groups was significant (*P*<0.05). The statistical analysis results are shown in Table 2.

**Table 2:** HIV/AIDS awareness rate among 2734 male college students.

Variables	Casual sexual behavior group (*n* = 595)						Without casual sexual behavior group (*n* = 2139)						*x*^*2*^	*P*
	Correct	%	Wrong	%	Don’t know	%	Correct	%	Wrong	%	Don’t know	%		
Item 1	462	77.65	105	17.65	28	4.7	1608	75.18	469	21.92	62	2.9	9.055	0.011
Item 2	294	49.41	240	40.34	61	10.25	929	43.43	963	45.02	247	11.55	6.737	0.034
Item 3	444	74.62	87	14.62	64	10.76	1894	88.55	101	4.72	144	6.73	86.81	<0.001
Item 4	457	76.81	104	17.48	34	5.71	1930	90.23	129	6.03	80	3.74	85.55	<0.001
Item 5	548	92.1	25	4.2	22	3.7	2082	97.34	30	1.4	27	1.26	34.86	<0.001
Item 6	540	90.76	30	5.04	25	4.2	2056	96.12	44	2.06	39	1.82	27.98	<0.001
Item 7	376	63.19	104	17.48	115	19.33	1363	63.72	334	15.61	442	20.67	1.438	0.487
Answering 5 or more items correctly	477	80.17	118	19.83			1851	86.54	288	13.46			14.93	<0.001

### Analysis of factors influencing casual sexual behavior

In the univariate analysis, the participants who had received the school’s HIV test promotion in the last year (*Crude OR* = 1.48), those who had received the self-assessment of AIDS risk conducted by the school (*Crude OR* = 1.98), those who were aware that the school had HIV self-test kits available (*Crude OR* = 3.04), those who had accepted a one-night stand (*Crude OR* = 4.29), those who had accepted commercial sex (*Crude OR* = 3.82), those who were MSM (*Crude OR* = 3.30), those who had heard of voluntary counseling and testing for AIDS (*Crude OR* = 1.49), and those who had accepted HIV voluntary counseling and testing services in the past year (*Crude OR* = 2.26) were more likely to engage in casual sexual behaviors. The participants who knew that the CDC provides HIV testing services (*Crude OR* = 0.63) and knew about HIV/AIDS prevention and treatment (*Crude OR* = 0.63) were more likely not to have casual sex. The statistical analysis results are shown in Table 3.

**Table 3:** Analysis of factors influencing casual sexual behavior among male college students.

Variables	Casual sexual behavior group (n = 595)		Without casual sexual behavior group (n = 2139)		Univariate analysis		Multivariate analysis	
	*n*	*%*	*n*	*%*	*Crude OR* (95%*CI*)	*P*	*Ajusted OR* (95%*CI*)	*P*
**Age (years)**								
≤19	196	32.9	631	29.5	1.0		1.0	
20–21	309	51.9	1135	53.1	1.14(0.93–1.40)	0.205	1.11(0.84–1.47)	0.481
≥22	90	19.4	373	17.4	1.29(0.97–1.70)	0.078	1.33(0.90–1.95)	0.152
**Hometown**								
City	166	27.9	518	24.2	1.0		1.0	
Town	109	18.3	292	13.7	0.75(0.61–0.93)	0.009	1.27(0.93–1.73)	0.131
Rural area	320	53.8	1329	62.1	0.65(0.50–0.83)	0.001	0.86(0.68–1.09)	0.201
**Grades**								
Freshman	121	20.3	405	18.9	1.0		1.0	
Sophomore	200	33.6	642	30	1.04(0.81–1.35)	0.75	0.87(0.65–1.18)	0.371
Junior	206	34.6	674	31.5	1.02(0.79–1.32)	0.862	0.81(0.57–1.15)	0.24
Senior	68	11.4	418	19.5	0.55(0.39–0.76)	<0.001	0.46(0.30–0.71)	<0.001
**Whether had known about HIV/AIDS prevention and treatment**								
No	118	19.8	288	13.5	1.0		1.0	
Yes	477	80.2	1851	86.5	0.63(0.50–0.80)	<0.001	0.66 (0.51–0.86)	<0.001
**Whether received a school lecture on HIV/AIDS in the past year**								
No	178	29.9	713	33.3	1.0		——	
Yes	417	70.1	1426	66.7	1.17(0.96–1.43)	0.116		
**Whether had received the school’s HIV test promotion in the last year**								
No	185	31.1	855	40	1.0		1.0	
Yes	410	68.9	1284	60	1.48(1.22–1.79)	<0.001	1.09(0.86–1.39)	0.465
**Whether had received the self-assessment of AIDS risk conducted by the school**								
No	296	49.7	1417	66.2	1.0		1.0	
Yes	299	50.3	722	33.8	1.98(1.65–2.38)	<0.001	1.45(1.14–1.84)	0.003
**Whether had awared that the school had HIV self-test kits available**								
No	392	65.9	1828	85.5	1.0		1.0	
Yes	203	34.1	311	14.5	3.04(2.47–3.75)	<0.001	2.02(1.56–2.62)	<0.001
**Whether had accepted a one-night stand**								
No	135	22.7	1192	55.7	1.0		1.0	
Yes	460	77.3	947	44.3	4.29(3.48–5.29)	<0.001	2.82(2.18–3.66)	<0.001
**Whether had accepted commercial sex**								
No	260	43.7	1599	74.8	1.0		1.0	
Yes	335	56.3	540	25.2	3.82(3.16–4.61)	<0.001	1.95(1.53–2.48)	<0.001
**Whether had accepted homosexual sex between MSM**								
No	456	76.6	1958	91.5	1.0		1.0	
Yes	139	23.4	181	8.5	3.30(2.59–4.21)	<0.001	1.81(1.37–2.39)	<0.001
**Whether had heard of voluntary counseling and testing for AIDS**								
No	238	40	1067	49.9	1.0		1.0	
Yes	357	60	1072	50.1	1.49(1.24–1.80)	<0.001	1.14(0.90–1.43)	0.276
**Whether had known that the CDC provides HIV testing services**								
No	85	14.3	202	9.4	1.0		1.0	
Yes	510	85.7	1937	90.6	0.63(0.48–0.82)	0.001	0.56(0.41–0.77)	<0.001
**Whether had accepted HIV voluntary counseling and testing services in the past year**								
No	534	89.7	2036	95.2	1.0		1.0	
Yes	61	10.3	103	4.8	2.26(1.62–3.14)	<0.001	1.34(0.91–1.97)	0.14
Condom use self-efficacy measurement^*^								
No confidence	26	4.7	90	4.3	1.0		——	
Have confidence	147	26.5	724	34.9	0.70(0.44–1.13)	0.142		
Very confident	381	68.8	1260	60.8	1.05(0.67–1.64)	0.843		
Whether had sex with a regular partner in the past year^*^								
Yes	400	70.4	1381	67.4	1.0		——	
No	168	29.6	668	32.6	1.15(0.94–1.41)	0.172		
Whether had condom use during sex with a fixed partner^*^								
Never use	117	29.3	67	4.9	1.0		——	
Sometimes/frequently used	154	38.6	510	37.2	0.17(0.12–0.25)	<0.001		
Always use	128	32.1	795	57.9	0.09(0.07–0.13)	<0.001		

*There is missing data.

In the multivariate analysis, there were no significant differences in the two demographic characteristics of age and hometown (*P*>0.05). The participants who had received a self-assessment of HIV risk conducted by the school (*Ajusted OR* = 1.45, 95% *CI* = 1.14–1.84), those who were aware HIV self-test kits were available at school (*Ajusted OR* = 2.02, 95% *CI* = 1.56–2.62), those who had accepted a one-night stand (*Ajusted OR* = 2.82, 95% *CI* = 2.18–3.66), those who had accepted commercial sex (*Ajusted OR* = 1.95, 95% *CI* = 1.53–2.48), and those who were MSM (*Ajusted OR* = 1.81, 95% *CI* = 1.37–2.39) were more inclined to have casual sex. The participants who were seniors (*Ajusted OR* = 0.46, 95% *CI* = 0.30–0.71), those who had awareness of HIV/AIDS prevention and treatment (*Ajusted OR* = 0.66, 95% *CI* = 0.51–0.86), and those who knew that the CDC provides HIV testing services (*Ajusted OR* = 0.56, 95% *CI* = 0.41–0.77) were not inclined to engage in casual sexual behaviors.

## Discussion

This was a cross-sectional survey of students conducted in colleges and universities in Zhejiang Province of China, which can reflect the current situation of casual sexual behaviors among college male students. The results of this study revealed that the proportion of male college students engaging in casual sex was 21.8%, which shows that the frequency of casual sexual behaviors in this group is relatively high. The key protective measures for casual sexual behaviors include the following. To begin with, every person needs to improve sexual responsibility and morality, and reduce the frequency of sexual behaviors with temporary partners. In addition, it is necessary to enhance self-prevention awareness and ability, insist on using condoms, and emphasize knowingly making friends. Studies have shown that increasing the population’s awareness of HIV/AIDS and promoting behavioral changes are important means to protect susceptible populations [16, 17].

Receiving the HIV risk self-assessment conducted by the school and awareness that HIV self-testing kits were available at school were influencing factors, and knowledge of HIV/AIDS prevention and treatment was a protective factor against casual sexual behavior among male college students. Studies have shown that increasing the population’s HIV/AIDS awareness rate and promoting consistent knowledge and behaviors are important means to protect susceptible populations [18, 19]. It is necessary to further strengthen campus health education and increase publicity to promote the integration of knowledge and actions to prevent HIV infection.

This study found that the proportion of male college students in the casual sexual behavior group who were accepting of one-night stands and commercial sex and who were MSM was higher than that in the group without casual sexual behavior, suggesting that the sexual attitudes of the casual sexual behavior group were more open. At the same time, being a senior as opposed to a freshman was a protective factor against casual sexual behavior among male college students because senior male college students may be busy with graduation and finding employment, in contrast to sophomore/junior/freshman male college students. Previous studies have shown that a higher degree of openness in sexual attitudes is more likely to occur with multiple sexual partners, that family sex education plays a significant role in reducing risky sexual behaviors, and that providing earlier HIV prevention education serves as a protective factor against college students’ sexual behavior [3, 20, 21]. Therefore, it is necessary to provide sex education correctly and early, to encourage family sex education, and to guide students to establish the correct sexual concepts and attitudes.

In the multifactor analysis, the results showed that having received the HIV risk self-assessment conducted by the school, knowing that HIV self-test kits are available at the school, and being accepting of one-night stands, commercial sex, and sex with other men were five risk factors affecting the casual sexual behavior of male college students. Knowing that the CDC provides HIV testing services, having HIV/AIDS prevention knowledge, and being a senior were three protective factors against male college students having casual sex.

This study had the following shortcomings. First, this study was a cross-sectional survey, there may have been recall bias, and causal inferences could not be made about the influencing factors. In addition, the questionnaire was not designed specifically for the casual sexual behaviors of male college students. The content of the survey was self-reported by the participants, which may be biased.

## Conclusions

Male college students who engage in casual sexual behaviors have a high degree of openness in sexual attitudes, lack of knowledge about HIV prevention, and knowledge of testing-related information but low testing rates. It is recommended that corresponding health education programs be formulated based on the characteristics of this population. It is important to strengthen the publicity of HIV testing-related knowledge, increase sex education, and increase the risk awareness of college students, thereby increasing the HIV testing rate and reducing AIDS-related harm to college students.

## Supporting information

S1 Questionnaire Chinese version of the questionnaire. (DOCX)

S2 Questionnaire English version of the questionnaire. (DOCX)

S1 File (RAR)

## Abbreviations

HIV: Human immunodeficiency virus
AIDS: Acquired Immune Deficiency Syndrome
STDs: Sexually transmitted diseases
MSM: Men who have sex with men
CDC: Center for Disease Control and Prevention
