# Supplementary material for: Analysis of factors influencing casual sexual behavior among male college students in Zhejiang Province, China
Source: PLoS One. 2021 May 3;16(5):e0250703. doi: 10.1371/journal.pone.0250703 (PMC8092760; doi:10.1371/journal.pone.0250703)
Supplement: S1 Questionnaire — (DOCX) [file pone.0250703.s001.docx]

大学生艾滋病相关知识、态度和行为基线调查问卷

为保障大学生的身体健康，省疾病预防控制中心组织开展本次调查，以了解大学生艾滋病相关知识和行为，为制定大学生艾滋病健康教育策略提供科学依据。

本次调查采用不记名形式，对于您参加的本次调查将严格保密，您的任何信息都不会告知学校。调查大约会占用您10分钟的时间。请您仔细阅读问卷后，根据个人的情况填写，保证内容真实准确，为大学生的健康贡献您的一份力量。

学校： 学院： 专业：

年级： 班级：

**一、基本情况**

1. 性别：①男②女
2. 年龄（周岁）：
3. 户籍所在地：省市
4. 户籍：①城市②城镇③农村
5. 每个月的生活费：元
6. 关于您的家庭：①父母关系很好②,父母关系一般③父母关系不好

④父母已离婚

**二、艾滋病知识**

| 7. 艾滋病是一种不可治愈的严重传染病 | ①正确 ②错误 ③不知道 |
| --- | --- |
| 8. 目前我国青年学生中艾滋病主要传播方式为男男同性性行为 | ①正确 ②错误 ③不知道 |
| 9. 通过外表可以判断一个人是否感染艾滋病 | ①正确 ②错误 ③不知道 |
| 10. 日常生活和学习接触会传播艾滋病 | ①正确 ②错误 ③不知道 |
| 11. 坚持正确使用安全套可以减少感染和传播艾滋病的风险 | ①正确 ②错误 ③不知道 |
| 12. 发生高危性行为后（如多性伴/不使用安全套等），应主动寻求艾滋病咨询与检测 | ①正确 ②错误 ③不知道 |
| 13. 国家为HIV感染者/病人提供免费抗病毒治疗 | ①正确 ②错误 ③不知道 |

**三、宣传教育**

14. 最近一年您接受过学校开展的艾滋病专题讲座或健康教育课吗？

①是②否

15. 最近一年您有通过学校的网络媒体（公众号、QQ群、APP等）了解到艾滋病信息

吗？①有②没有

16. 最近一年您参加过学校开展的下列哪些艾滋病宣传活动？**（可多选）**

①主题班会②知识竞赛③绘画竞赛④读者日活动⑤辩论赛⑥情景剧比赛

⑦微作品（微电影、微小说等）征集⑧课件设计大赛⑨艾滋病日主题方案征集

⑩纪录片制作、宣传栏、校园广播、校园网、图片展览

17. 最近一年您是否接受过学校关于艾滋病检测的宣传？

①是②否**（跳转至第18题）**

17.1如果有，主要是哪些方面的宣传？**（可多选）**

①艾滋病检测机构②艾滋病检测的作用③艾滋病自愿咨询检测④艾滋病快速检测⑤艾滋病自我检测⑥艾滋病窗口期

⑦其他（请注明）

18. 您有没有接受过学校开展的艾滋病风险自我评估？①有②没有

19. 您所在的学校是否有HIV自我检测试剂售卖或领取？①有②无③不知道

**四、性态度**

20. 您能接受一夜情吗？①接受②不接受③不知道

21. 您能接受商业性行为（有金钱交易，如包养、嫖娼、卖淫）吗？

①接受②不接受③不知道

22. 您能接受男男同性性行为吗？①接受②不接受③不知道

**五、性行为**

23.到目前为止，您是否发生过性行为？①是②否（**跳转至第40题**）

24到目前为止，您的性行为对象：①同性②异性③两者都有

25. 最近一年，您是否与固定性伴（如男/女朋友）发生性行为？

①是②否**（跳转至第28题）**

26. 最近一年，您的固定性伴来自于哪个人群？**（可多选）**

①本校学生②外校学生③社会人员

27.最近一年，您与固定性伴发生性行为时是否使用安全套？

①从不使用②有时使用③经常使用④每次都使用

28最近一年，您是否发生过临时性行为（如一夜情/约炮、与一般熟人的性行为）？

①是②否**（跳转至第40题）**

29. 最近一年您与个人发生临时性行为？

30. 最近一年，您的临时性伴来自于哪个人群？**（可多选）**

①本校学生②外校学生③社会人员

31.最近一年，您通过哪些途径寻找过临时性伴？**（可多选）**

①手机交友软件②娱乐场所③网络游戏

④互联网直播平台⑤一般熟人⑥其他

32. 您通过以下哪个手机交友软件寻找过临时性伴？**（可多选）**

①未使用手机交友软件寻找性伴②微信③陌陌④探探

⑤Blued ⑥Zank ⑦Jacked ⑧Aloha ⑨其他

33.最近一年，您饮酒后是否发生过临时性行为？①是②否

34.最近一年，您与临时性伴发生性行为时是否使用安全套？

①从不使用②有时使用③经常使用④每次都使用

35. 最近一年，您与临时性伴发生性行为前或过程中是否讨论过安全套使用？

①是②否

36. 您是否被临时性行为对象要求尝试肛交性行为? ①是②否

37.最近一年发生临时性行为前，您是否想知道对方艾滋病感染状况？①是②否③没想过

38. 最近一年，您是否发生过有金钱交易的商业性行为（如嫖娼）？①是②否

39.您觉得自己有感染艾滋病的风险吗？①有②没有③不知道

40. 安全套使用效能

(1). 发生性行为前您有信心和性伴讨论安全套使用吗？

①非常有信心②很有信心③有信心④没有信心⑤非常没信心

(2)如果性行为时您的性伴不同意使用安全套或未携带安全套，您有信心不发生性行为吗？

①非常有信心②很有信心③有信心④没有信心⑤非常没信心

(3). 如果发生性行为您有信心提前购买安全套吗？

①非常有信心②很有信心③有信心④没有信心⑤非常没信心

**六、艾滋病检测利用和意愿**

41.您是否听说过以下艾滋病检测服务？

| 检测服务 | 是否听说过 |
| --- | --- |
| (1)艾滋病自愿咨询检测（VCT） | ①是②否 |
| (2)HIV自我检测 | ①是②否 |
| (3)HIV网络预约检测 | ①是②否 |
| (4)HIV血液（指尖血/静脉血）快速检测 | ①是②否 |
| (5)HIV口腔唾液快速检测 | ①是②否 |
| (6)HIV尿液检测 | ①是②否 |

42.最近一年，您接受过几次以下检测服务（请填写具体次数，若无，请填“0”）。

| 检测服务 | 接受HIV检测次数 |
| --- | --- |
| (1)艾滋病自愿咨询检测（VCT） | 次 |
| (2)HIV自我检测 | 次 |
| (3)HIV网络预约检测 | 次 |
| (4)HIV血液（指尖血/静脉血）快速检测 | 次 |
| (5)HIV口腔唾液快速检测 | 次 |
| (6)HIV尿液检测 | 次 |

43.您知道以下机构是否提供HIV检测服务？

| 检测项目 | 是否提供HIV检测服务 |
| --- | --- |
| (1) 疾病预防控制中心 | ①是②否③不清楚 |
| (2) 综合性医院 | ①是②否③不清楚 |
| (3) 社区卫生服务中心 | ①是②否③不清楚 |
| (4) 校医院/医务室等 | ①是②否③不清楚 |
| (5)社会组织(包括社区小组) | ①是②否③不清楚 |

44.最近一年，您接受过几次以下机构提供的HIV检测服务（请填写具体次数，若无，请填写“0”）。

| 检测项目 | 接受HIV检测次数（若无，填“0”） |
| --- | --- |
| (1) 疾病预防控制中心 | 次 |
| (2) 综合性医院 | 次 |
| (3) 社区卫生服务中心 | 次 |
| (4) 校医院/医务室等 | 次 |
| (5)社会组织(包括社区小组) | 次 |

**调查结束，谢谢！**
