# Supplementary material for: Analysis of factors influencing casual sexual behavior among male college students in Zhejiang Province, China
Source: PLoS One. 2021 May 3;16(5):e0250703. doi: 10.1371/journal.pone.0250703 (PMC8092760; doi:10.1371/journal.pone.0250703)
Supplement: S2 Questionnaire — (DOCX) [file pone.0250703.s002.docx]

**Baseline survey questionnaire on AIDS-related knowledge, attitudes and behaviors of college students**

*In order to protect the health of college students, the Zhejiang Provincial Center for Disease Control and Prevention organized this survey to understand college students’ AIDS-related knowledge and behaviors and provide a scientific basis for formulating college students’ AIDS health education strategies.This survey is in an anonymous form. The survey you participated in will be kept strictly confidential, and no information about you will be notified to the school. The survey will take about 10 minutes of your time. Please read the questionnaire carefully and fill it out according to your personal circumstances to ensure that the content is true and accurate, and contribute your strength to the health of college students.*

School: __________ College: __________ Major: __________

Grade: __________ Class: __________

**Basic situation**

1. Gender: (1)Male (2)Female

2. Age (years old): __________

3. Place of household registration: Province__________ City__________

4. Household registration: (1)Urban (2)Township (3)Rural

5. Monthly living expenses: __________ yuan(RMB)

6. Regarding your family: (1)Parent relationship is very good (2)Parent relationship is average (3)Parent relationship is not good (4)Parents are divorced

**AIDS knowledge**

7. AIDS is a serious and incurable infectious disease (1)Correct (2)Incorrect (3)Don’t know

8. At present, the main mode of HIV transmission among young students in my country is male homosexual sex. (1)Correct (2)Incorrect (3)Don’t know

9. You can judge whether a person is infected with HIV by appearance (1)Correct (2)Incorrect (3)Don’t know

10. Daily life and study contact can transmit HIV (1)Correct (2)Incorrect (3)Don’t know

11. Adhere to the correct use of condoms can reduce the risk of infection and transmission of HIV (1)Correct (2)Incorrect (3)Don’t know

12. After high-risk sexual behavior (such as multiple sexual partners/not using condoms, etc.), you should actively seek HIV counseling and testing. (1)Correct (2)Incorrect (3)Don’t know

13. The state provides free antiretroviral treatment for HIV-infected/patients (1)Correct (2)Incorrect (3)Don’t know

**Publicity and education**

14. Have you received any AIDS seminars or health education classes offered by your school in the past year? (1)Yes (2)No

15. In the past year, have you learned about HIV/AIDS through the school’s online media (public account, QQ group, APP, etc.) ? (1)Yes(2)No

16. In the past year, which of the following AIDS publicity activities have you participated in? (Multiple choice) (1)Themed class meeting (2)Knowledge contest (3)Painting contest (4)Reader's day event (5)Debate contest (6)Situation contest (7)Call for micro works (micro movies, micro talks, etc.) (8)Courseware design competition (9)Call for AIDS Day theme plans (10) Documentary production, publicity column, campus broadcast, campus network, photo exhibition

17. Have you received any publicity about HIV testing in your school in the past year? (1)Yes (2)No (skip to question 18)

17.1 If yes, what are the main areas of publicity? (Multiple choice)

(1) AIDS testing institutions (2) The role of HIV testing (3) Voluntary HIV counseling and testing (4)HIV rapid test (5)HIV self-test (6)HIV window period (7)Others (please specify)

18. Have you ever received an AIDS risk self-assessment conducted by your school? (1)Yes (2)No

19. Does your school sell or receive HIV self-test kits? (1) Yes (2) No (3) Don’t know

**Sexual attitude**

20. Can you accept a one-night stand? (1)Accept (2)Don't accept (3)Don’t know

21. Can you accept commercial sex (money transactions, such as keep a mistress, whoring, prostitution)? (1)Accept (2)Don't accept (3)Don’t know

22. Can you accept gay sex? (1)Accept (2)Don't accept (3)Don’t know

**Sexual behavior**

23. Have you had sex so far? (1)Yes(2)No (skip to question 40)

24 So far, your sexual objects: (1)same sex (2)heterosexual (3)both

25. In the past year, have you had sex with a regular partner (such as a boy/girlfriend)? (1)Yes (2)No (skip to question 28)

26. In the past year, from which group did your regular sexual partner come from? (Multiple choice) (1)Students of the school (2)Students of other schools (3)Social staff

27. In the past year, did you use condoms when you had sex with a regular partner? (1)Never use (2)Sometimes (3)Use often (4)Use every time

28 In the past year, have you ever had temporary sex (such as one-night stand/appointment, sex with ordinary acquaintances)? (1)Yes (2)No (skip to question 40)

29. Have you had temporary sex with an individual in the past year?

30. In the past year, from which group did your temporary sexual partner come from? (Multiple choice) (1)Students of the school (2)Students of other schools (3)Social staff

31. In the past year, how did you find temporary sexual partners? (Multiple choice) (1)Mobile dating software(2)Entertainment place(3)Online game

(4)Internet live broadcast platform (5)General acquaintance (6)Other

32. Which of the following mobile dating apps have you used to find temporary sex partners? (Multiple choice) (1)Do not use mobile dating software to find sexual partners (2)WeChat (3)Momo (4)Tantan (5)Blued (6)Zank (7)Jacked (8)Aloha (9)Other

33. In the past year, have you ever had temporary sex after drinking alcohol? (1)Yes(2)No

34. In the past year, did you use a condom when you had sex with a temporary partner? (1)Never use (2)Sometimes (3)Use often (4)Use every time

35. In the past year, have you discussed condom use before or during sex with your temporary partner? (1)Yes (2)No

36. Have you been asked to try anal sex by a temporary sex object? (1)Yes (2)No

37. Before you had temporary sex in the last year, did you want to know the status of your partner's HIV infection? (1)Yes (2)No (3)Never thought about it

38. In the past year, have you ever had any commercial behaviors involving money transactions (such as prostitution)? (1)Yes (2)No

39. Do you think you are at risk of contracting AIDS? (1)Yes (2)No (3)Don’t know

40. Condom use effectiveness

(1). Do you have the confidence to discuss condom use with your partner before having sex? (1)very confident (2)very confident (3)confident (4)not confident (5)very unconfident

(2) If your partner does not agree to use a condom or does not carry a condom during sex, are you confident that you will not have sex? (1)very confident (2)very confident (3)confident (4)not confident (5)very unconfident

(3). If you have sex, are you confident to buy condoms in advance? (1)very confident (2)very confident (3)confident (4)not confident (5)very unconfident

**HIV testing utilization and willingness**

41. Have you heard of the following HIV testing services?

| Inspection Service | Have you heard of this testing service |
| --- | --- |
| (1) AIDS Voluntary Counseling and Testing (VCT) | (1)Yes (2)No |
| (2) HIV self-test | (1)Yes (2)No |
| (3) HIV online appointment testing | (1)Yes (2)No |
| (4) HIV blood (fingertip blood/venous blood) rapid test | (1)Yes (2)No |
| (5) HIV oral saliva rapid test | (1)Yes (2)No |
| (6) HIV urine test | (1)Yes (2)No |

42. In the past year, you have received several of the following testing services (please fill in the specific frequency, if none, please fill in "0").

| Inspection Service | Number of received HIV tests |
| --- | --- |
| (1) AIDS Voluntary Counseling and Testing (VCT) |  |
| (2) HIV self-test |  |
| (3) HIV online appointment testing |  |
| (4) HIV blood (fingertip blood/venous blood) rapid test |  |
| (5) HIV oral saliva rapid test |  |
| (6) HIV urine test |  |

43. Do you know whether the following agencies provide HIV testing services?

| Test items | Whether to provide HIV testing services |
| --- | --- |
| (1) Center for Disease Control and Prevention | (1)Yes(2)No(3)Uncertain |
| (2) General hospital | (1)Yes(2)No(3)Uncertain |
| (3) Community Health Service Center | (1)Yes(2)No(3)Uncertain |
| (4) School hospital/infirmary, etc. | (1)Yes(2)No(3)Uncertain |
| (5) Social organizations (including community groups) | (1)Yes(2)No(3)Uncertain |

44. In the past year, you have received several HIV testing services provided by the following institutions (please fill in the specific number of times, if none, please fill in "0").

| Test items | Number of received HIV tests (if none, please fill in "0") |
| --- | --- |
| (1) Center for Disease Control and Prevention |  |
| (2) General hospital |  |
| (3) Community Health Service Center |  |
| (4) School hospital/infirmary, etc. |  |
| (5) Social organizations (including community groups) |  |

The investigation is finished, thank you!
